# Nanostructured mesoporous silica: influence of the preparation conditions on the physical-surface properties for efficient organic dye uptake

**DOI:** 10.1098/rsos.172021

**Published:** 2018-03-14

**Authors:** Rania E. Morsi, Rasha S. Mohamed

**Affiliations:** 1 Egyptian Petroleum Research Institute, Nasr City, P.B. 11727, Cairo, Egypt; 2 EPRI-Nanotechnology Center, Egyptian Petroleum Research Institute, Nasr City, PO Box 11727, Cairo, Egypt

**Keywords:** ordered mesoporous silica, methylene blue dye uptake, kinetics, isotherms, column flow system

## Abstract

A series of ordered mesoporous silica such as MCM-41, SBA-3 and SBA-15, in addition to silica micro- (SM) and nano- (SN) mesoporous particles, were prepared. The preparation conditions were found to greatly influence the physical-surface properties including morphological structure, porosity, particle size, aggregate average size, surface area, pore size, pore volume and zeta potential of the prepared silica, while the chemical structure, predicted from FT-IR spectra, and the diffraction patterns, predicted from wide-angle X-ray diffraction spectra, were identical. Surface areas of approximately 1500, 1027, 600, 552 and 317 m^2^ g^−1^, pore volumes of 0.93, 0.56, 0.82, 0.72 and 0.5 cm^3^ g^−1^, radii of 2.48, 2.2, 5.66, 6.6 and 8.98 nm, average aggregate sizes of 56, 65.4, 220.9, 73, 61.1 and 261 nm and zeta potential values of −32.8, −46.1, −26.3, −31.4 and −25.9 mV were obtained for MCM-41, SBA-3, SBA-15, SN and SM, respectively. Methylene blue dye uptake capacity of the prepared silica types was investigated using the batch technique and, in addition, the most effective material was further studied by the column flow system. The kinetics and isotherms of the uptake process were studied. The morphological structure, surface area, pore radius and zeta potential values were the most correlated factors.

## 1. Introduction

Recently, there has been a growing interest in nanomaterials. The properties of materials with nanometre dimensions are significantly different from those of bulk materials because of (i) large fraction of surface atoms; (ii) high surface energy; (iii) spatial confinement and (iv) reduced imperfections [1–4].

Porous materials are solids that contain empty pores dispersed within their framework [5]. The pores may be open pores connecting to the outside of the material or closed pores isolated from the outside. Porous materials have lower density and higher surface area compared with dense materials. According to the IUPAC definition, porous materials are divided into three types based on their pore diameters (*d*); microporous with *d* < 2 nm, mesoporous with *d* = 2–50 nm and macroporous with *d *> 50 nm [6].

Mesoporous materials are an important class of nanostructured materials that possess high specific surface area, large pore volume and rich surface chemistry [7]. They recently have witnessed an exponential growth in research and development and in innumerable applications such as catalysis (as catalysts or supports) [8], adsorption [9], pollutant remediation [10], sensors [11], drug delivery systems [12], photocatalysis [13], solar cells [14], fuel cells and batteries [15,16]. Mesoporous materials can have a wide range of compositions but mainly consist of oxides such as SiO_2_, TiO_2_, ZnO_2_, Fe_2_O_3_ or combinations of metal oxides, but also mesoporous carbons can be synthesized [17–20].

Mesoporous silica materials represent a unique class of silica-based materials that possess high specific surface area, large pore volume and a uniform pore size between 2 and 50 nm [21]. Mesoporous silica can be synthesized in both basic and acidic environments and typically relies on a surfactant template to produce its mesostructures. The breakthrough came in 1992 with the discovery of M41S family of mesoporous silica which possess various physico-chemical properties such as high surface area (greater than 1000 m^2^ g^−1^), tuneable pore size (20–100 Å), large pore volumes (greater than 0.6 cm^3^ g^−1^), thermal stability up to 800°C in dry atmosphere and higher hydrothermal stability. They are also characterized with large channels, from 1.5 to 10 nm, ordered in a hexagonal, cubic or lamellar array. MCM-41 is the most important member of the M41S family, with *p*6mm symmetry space group [17–20,22,23]. On the other hand, SBA-X family (Santa Barbara Amorphous), where *X* is a number corresponding to a specific pore structure and surfactant (SBA-11, SBA-12, SBA-15 and SBA-16), were synthesized in acidic medium using non-ionic surfactants having polyethylene oxide units as reported in 1998 by Zhao *et al*. [24–26], while many framework structures, such as SBA-1, SBA-2 and SBA-3, were synthesized using cationic surfactants [27,28].

Each family has its own unique advantages and disadvantages, and all have been successfully used in various applications. These applications include excellent candidates for drug delivery, biomedical, diagnostic, medical imaging, engineering catalyst supports, adsorption, separation of proteins, cell imaging, cell labelling, enzyme adsorption and immobilization with good biocompatibility [29–37].

The properties of mesoporous silica allow higher loading of drugs or biomolecules, improved control over the loading and release kinetics, and higher biocompatibility because it is easy for it to be chemically modified. On the other hand, mesoporous silica has been used to adsorb heavy metal ions [38,39], organic dyes [40], polycyclic aromatic hydrocarbons [41] and other organic pollutants, and the results indicate that the mesoporous silica has excellent adsorption ability [42].

Organic pollutants soluble in water are one of the most dangerous pollutants which may make environmental conditions more toxic and even more carcinogenic [43]. Exposure to low concentrations of some of these molecules or their degradation products may cause a lot of diseases. An effective purification active material should be able to remove contaminants to the standard limits before discharging wastewaters to aquatic ecosystems or reuse in industry. Among the treatments proposed, adsorption is a well-known equilibrium separation process. It is now recognized as an effective, efficient and economic method [29–31,44,45].

Among the numerous published works on the different families of silica nanomaterials, there are scant studies dealing with comparisons between the different preparation conditions and the physical-surface properties of the produced materials. Also, the information related to correlations between these properties and the uptake capacities of these silica materials is few when compared with the expected useful information that could be obtained. In this work, a series of silica nanomaterials were prepared under different conditions, and the physical-surface properties of the produced materials were correlated to the preparation conditions. The uptake capacity of methylene blue dyes using the prepared materials was compared and correlated to their properties, and the uptake kinetics and isotherms studies were represented.

## 2. Experimental part

### 2.1. Materials and reagents

Triblock copolymer Pluronic P123 (PEG 20-PPG 70-PEG 20) poly(ethylene glycol)-block-poly(propylene glycol)-block-poly(ethylene glycol) molecular weight of 5800 and tetraethyl orthosilicate (TEOS) were purchased from Sigma-Aldrich, and cationic surfactant cetyltrimethyl ammonium bromide (CTAB) was purchased from MP Biomedicals, Inc., France. Thirty per cent solution of ammonium hydroxide, 37% hydrochloric acid, 98% acetic acid, absolute ethanol and methylene blue (MB) were purchased from Sigma Chemicals. All other chemicals and reagents were of analytical grade and were used as received without further purifications.

### 2.2. Material synthesis

#### 2.2.1. Silica microparticles

The synthesis of silica microparticles (SM) was carried out according to the method described by Stöber *et al*. [32] and developed by other authors [33–37]. Briefly, 14.4 ml absolute ethanol, 18 ml of water and 8 ml of 30% aqueous ammonia were added in 100 ml three-neck reaction flask equipped with a condenser and stirred at 55°C, and then 4 ml of TEOS was added to the solution. After 6 h at 55°C, the silica particles were separated from the solution by centrifugation and washed with water and ethanol. The produced material was thermally treated to remove the organic surfactant template using a programmable furnace where the temperature was raised at a rate of 2°C min^−1^ up to 550°C at which the sample was heated for further 6 h.

#### 2.2.2. Silica nanoparticles

Silica nanoparticles (SN) were prepared under basic conditions at room temperature as follows: to 300 ml of 0.82 mol l^−1^ ammonia solution (30%), 100 ml of ethanol was added with stirring followed by the addition of 16.7 ml of TEOS. The pH was adjusted to 10.0 using acetic acid-diluted solution and the final mixture was stirred for 5 h. Thereafter, the colloidal solution was separated by high-speed centrifuge (4000 r.p.m.), and the product was washed with distilled water and ethanol several times to remove undesirable particles followed by drying at 100°C for 2 h. The produced material was thermally treated to remove the organic surfactant template using a programmable furnace where the temperature was raised at a rate of 2°C min^−1^ up to 550°C at which the sample was heated for further 6 h [46–49].

#### 2.2.3. MCM-41

CTAB (8 g) was dissolved in 60 ml of deionized water with vigorous stirring for 2 h to get a clear solution which was then mixed with 100 ml of ethanol and 300 ml of 0.82 mol l^−1^ ammonia solution. TEOS (16.7 ml) was slowly added into the solution under rapid stirring and the pH was adjusted to 10.0 and stirring was continued at room temperature for 5 h. The product was recovered by centrifugation and washed several times with distilled water. The produced material was thermally treated to remove the organic surfactant template using a programmable furnace where the temperature was raised at a rate of 2°C min^−1^ up to 550°C at which the sample was heated for further 6 h [21,48–50].

#### 2.2.4. SBA-15

SBA-15 was synthesized according to the method described by Kim *et al*. [51]. Briefly, 8.0 g of triblock copolymer Pluronic P123 was dissolved in 240 g of 2 M HCl solution at 40°C and stirred for 2 h till a clear solution was obtained. TEOS weighing 16.7 g was added into the solution and stirred at 40°C for 24 h followed by ageing for 24 h at 40°C; the milky suspension thus obtained was transferred into a 500 ml polypropylene bottle and placed inside an oven dryer at 100°C for 24 h for hydrothermal treatment. Afterwards, the vessel was allowed to cool down. The suspension was filtered and washed with distilled water. The produced material was thermally treated to remove the organic surfactant template using a programmable furnace where the temperature was raised at a rate of 2°C min^−1^ up to 550°C at which the sample was heated for further 6 h [52–56].

#### 2.2.5. SBA-3

SBA-3 was prepared using 8 g of CTAB, as a template, dissolved in 240 g of HCl (2 M) with vigorous stirring at 40°C. After 2 h, 16.7 g of TEOS was added drop-wise to the acidic solution of CTAB with vigorous stirring at 40°C for 24 h [28]*.* The solution was transferred into a polypropylene bottle and heated at 80°C in an oven for 24 h. The resulting precipitate was filtered and washed with distilled water. The produced material was thermally treated to remove the organic surfactant template using a programmable furnace where the temperature was raised at a rate of 2°C min^−1^ up to 550°C at which the sample was heated for further 6 h [27,57–59].

### 2.3. Material characterization

The powder X-ray diffraction (XRD) patterns were recorded in both low angle (2*θ* range 0.5–10) and wide angle (2*θ* range 10–80) using a Bruker D8 advance X-ray diffractometer by Ni-filtered Cu K*α* radiation (*λ *= 1.54 Å); the accelerating voltage and applied current were 40 kV and 200 mA, respectively.

Nitrogen adsorption–desorption isotherms were measured at −196°C using a NOVA 3200 apparatus, USA. All samples were previously out-gassed to remove physically adsorbed water; liquid nitrogen was used during the nitrogen adsorption analysis under a reduced pressure at 300°C for 24 h. Specific surface areas were calculated using the Brunauer--Emmett--Teller (BET) model. Pore volumes are estimated at a relative pressure of (*P*/*P*_0_) range 0.05–0.30, assuming full surface saturation with nitrogen. Pore size distributions are evaluated from desorption branches of nitrogen isotherms using the Barrett--Joyner--Halenda (BJH) model [6].

FT-IR was used to reveal the chemistry of surface functional groups or to observe surface activation by AT1 Mattson model Genesis Series (USA) infrared spectrophotometer. The sample was firstly dried at 100°C and then was ground to fine powder in order to increase the homogeneity of the sample. In addition, the KBr technique was carried out approximately in a quantitative manner, as the weights of the sample and that of KBr were always kept constant. Transmission spectra were measured in the range of 4000–400 cm^−1^.

Thermo-gravimetric analyses (TGAs) were carried out for all samples using simultaneous DSC-TGA SDTQ 600, USA under N_2_ atmosphere, with a heating rate of 10°C min^−1^from room temperature up to 1000°C.

The average aggregate size of the nanoparticles was measured by dynamic light scattering particle sizing/zeta potential instrument technique, zetasizer nano series HT (Nano-ZS). The sample was dispersed in neutral water by sonication for 15 min, then measured in quartz cuvette.

The morphology of all samples was investigated with the aid of a JEOL TEM-1230 electron microscope, 120 kV, up to 600 000 magnifications, Japan. The samples were prepared by ultrasonic dispersion, using absolute alcohol as dispersing medium and then sonicated for 30 min to ensure a good dispersion. This solution was dropped on TEM carbon grids as support membranes.

Ultraviolet–visible spectrometer (JASCO V-630, Japan) was used to determine the concentration of MB with scanning in the range of 300–1100 nm.

### 2.4. Methylene blue uptake as an organic molecule model

#### 2.4.1. Batch experiments

Assessment of MB uptake is carried out by batch adsorption experiments performed in sealed glass flasks at room temperature. Pre-weighed amounts of the tested materials were added to glass flasks that contain the desired concentration of aqueous dye solution. The glass flasks were stirred for the desired time, separated and then the dye concentration was measured using UV–visible spectroscopy where a calibration curve was drawn at a wavelength of 640 nm (previously determined from a full scan of the MB solution). The effects of the adsorbent dosages (1, 5 and 10 g l^−1^), contact times (10–120 min) and the initial dye concentrations (10–100 ppm) were investigated. The amount of dye adsorbed was determined by the difference of the initial concentration (*C*_i_) and the equilibrium concentration (*C*_e_). The percentage of MB uptake was calculated as follows:

2.1 $$\text{Uptake}\;\%=\frac{C_{i}-C_{e}}{C_{i}}\times 100.$$

The adsorption capacity (*q*_e_) was calculated as follows:

2.2 $$q_{e}(\text{mg}\;{\text{g}}^{-1})=\frac{C_{i}-C_{e}}{M}\times V,$$

where *V* = volume of the MB solution (l) and *M* = weight of adsorbent (g).

More analysis of the experimental data was performed using common kinetic model equations to find the best-fitted model for the obtained data. The tested kinetic models are pseudo-first-order, first-order, pseudo-second-order and second-order kinetic models. In addition, the experimental adsorption data were fitted according to Langmuir and Freundlich adsorption isotherms models.

#### 2.4.2. Correlations of the physical-surface parameters and methylene blue uptake efficiency

Correlations of the physical-surface parameters of the prepared silica materials and the uptake efficiency at the same conditions (50 ppm initial MB concentration, 1 h contact time and 5 g l^−1^ adsorbent dose) were studied. The *R*^2^ values of the relation between the uptake capacity and the surface area; pore radius, pore volume, zeta potential and average aggregate size were used to determine the most effective property influencing the uptake efficiency.

#### 2.4.3. Flow uptake system

The flow uptake system was carried out in a glass column which contains MCM-41 (chosen according to the highest achieved MB uptake efficiency among the tested silica materials). Five hundred millilitres of 50 ppm MB solution was treated by flowing over 1 g of MCM-41 through the column with a diameter of 1 cm. The treated samples were collected in 5 ml intervals, and the remaining dye concentration in the solution was measured and the uptake efficiency was calculated as previously mentioned.

## 3. Results and discussion

### 3.1. Material characterization

**Figure 1.**
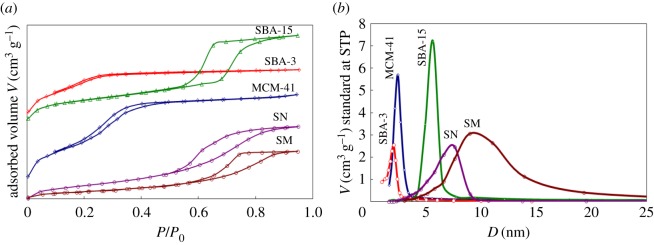
(*a*) N_2_ adsorption–desorption isotherms of silica samples and (*b*) pore size distribution curve of different prepared silica materials: MCM-41; SBA-3; SBA-15; SN and SM.

The nitrogen adsorption–desorption isotherms over the surface of the different silica materials as well as the pore size distribution curves of various prepared samples are illustrated in figure 1*a*,*b*, and their surface parameters are summarized in table 1. Three well-distinguished regions of the adsorption isotherms are evident: (i) monolayer–multilayer adsorption, (ii) capillary condensation and (iii) multilayer adsorption on the outer particle surface. Apparently, MCM-41 exhibits type IV isotherm with a distinct capillary condensation step, which is a characteristic pattern of mesoporous materials according to IUPAC classification. N_2_ adsorption–desorption isotherms of MCM-41 illustrate a clear H1-type hysteresis loop in the relative pressure range between 0.13 and 0.48, implying the presence of very regular mesoporous channels. For SBA-3, the isotherm is of type I according to IUPAC definition. Collapse of the hysteresis loop is indicated with pronounced narrowing of pore radius, indicating the presence of mesopores. SBA-15 showed the isotherm of type IV according to IUPAC classification being typical for mesoporous materials with H1 hysteresis loops in the relative pressure (*P*/*P*_0_) range of 0.54–0.89. Such type of loops can be referred to the presence of uniform cylindrical pores of relatively large dimensions. The isotherm of SN was of type IV, characteristic of mesoporous material according to IUPAC classification. The accompanying H2 hysteresis loop at *P*/*P*_0_ of 0.56–0.91 for SN is attributable to the ink-bottle pores of narrower orifice and border inner part. The isotherms of SM are type IV, which is a characteristic for mesoporosity with H4 hysteresis loop at *P*/*P*_0_ of 0.52–0.95 related to narrow slit pores.

**Table 1.** Surface parameters of different prepared silica materials: MCM-41, SBA-3, SBA-15, SN and SM.

materials	*S*_BET_ (m^2^ g^−1^)	*V*_P_ (cm^3^ g^−1^)	*r*_p_ (nm)
MCM-41	1499.5	0.929	2.48
SBA-3	1026.8	0.564	2.20
SBA-15	600.4	0.815	5.66
SN	552.1	0.721	6.60
SM	317.5	0.499	8.98

Based on these data in table 1, specific surface area (*S*_BET_) of 1499.5, 1026.8, 600.4, 552.1 and 317.5 m^2^ g^−1^ and total pore volume (*V*_p_) of 0.929, 0.564, 0.815, 0.721 and 0.499 cm^3^ g^−1^ were obtained for MCM-41, SBA-3, SBA-15, SN and SM, respectively. This is accompanied by the appearance of a group of narrower mesopores of most probable hydraulic pore diameter, *D* = 2.48, 2.20, 5.66, 6.60 and 8.98 nm, respectively, in the pore size distribution curves (figure 1*b*).

**Figure 2.**
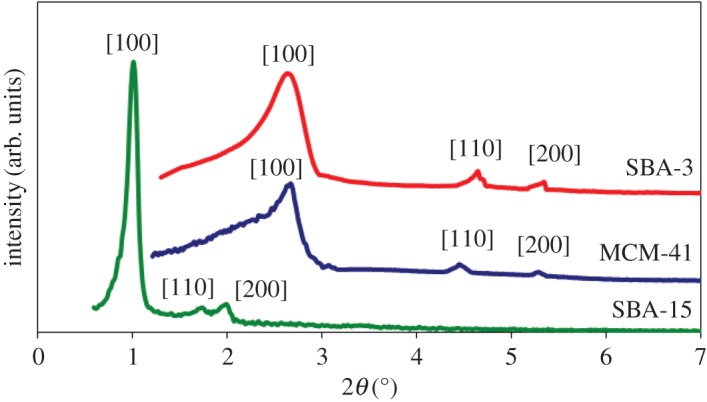
SAXRD patterns of ordered mesoporous silica samples: MCM-41; SBA-3 and SBA-15.

XRD is one of the most important techniques for characterizing the structure of crystalline or other ordered materials. In ordered porous MCM-41, SBA-3 and SBA-15 samples, the XRD peaks do not result from local order in the atomic range, but from the ordered channel walls. In general, ordered mesostructures can be demonstrated by low-angle XRD measurements. Diffraction X-ray patterns, obtained at small angles for the ordered mesoporous silica, are shown in figure 2. The small-angle XRD (SAXRD) pattern of MCM-41 has characteristic intense (100) peak and two obvious (110) and (200) reflections at 2*θ* ≈ 2.69, 4.5 and 5.4, respectively. The strong peak (100) directly indicates the presence of MCM-41 structure and the two weak peaks (110) and (200) can be indexed to the *P*6mm space group, indicating a hexagonal mesostructure material with a high degree of long-range ordering of the structure. SAXRD patterns of SBA-3 display three peaks at 2*θ* ≈ 2.5, 4.69 and 5.30, which are typical (100), (110) and (200) reflections of one-dimensional hexagonal (*P*6m) mesostructures and small-angle X-ray powder diffraction of SBA-15, three well-resolved peaks at 2*θ* ≈ 1.01, 1.77 and 2.01 are shown, being characteristic of the planes (100), (110) and (200), indicating a significant degree of long-range ordering in the structure and a well-formed two-dimensional hexagonal lattice.

**Figure 3.**
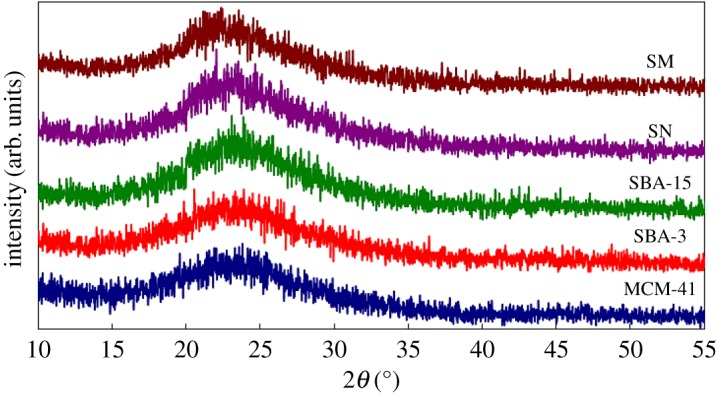
WAXRD patterns of the prepared silica samples: MCM-41; SBA-3; SBA-15; SN and SM.

Figure 3 shows the wide-angle X-ray powder diffraction (WAXRD) patterns of the prepared silica materials. A broad diffusion peak or an amorphous peak centred with the equivalent Bragg angle at 2*θ* = 23° was recorded, suggesting that the main substances of prepared silica MCM-41, SBA-3, SBA-15, SN and SM are amorphous silica phase.

**Figure 4.**
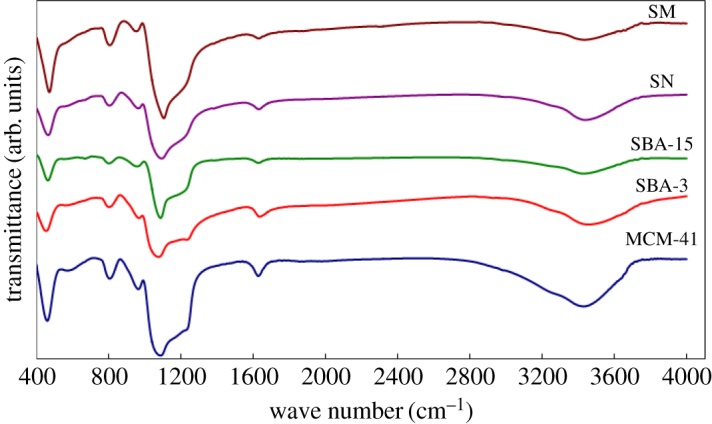
FT-IR spectra of silica samples: MCM-41; SBA-3; SBA-15; SN and SM.

The FT-IR spectra of the prepared materials are displayed in figure 4. Comparisons of the spectra show that they exhibit similar vibrational band characteristic of silica (SiO_2_). The broad band at 3460 cm^−1^ was attributed to the stretching vibration of H_2_O molecules. Correspondingly, the IR band at 1650 cm^−1^ is due to the bending vibration of H_2_O molecules. The shoulder at 3250 cm^−1^ could be assigned to the stretching vibrations of Si-OH groups in the structure of SiO_2_. The presence of the Si-OH group is proved as bonded water. The very strong and broad IR band at 1110 cm^−1^ with a shoulder at 1230 cm^−1^ is usually assigned to the longitudinal-optic (LO) and transverse-optic (TO) vibrational modes of the Si–O–Si asymmetric stretching vibrations. The IR band at 983 cm^−1^ was attributed to Si-OH bending (silanol groups). The IR band at 815 cm^−1^ can be assigned to SiO-H symmetric stretching vibrations, whereas the IR band at 470 cm^−1^ can be attributed to O–Si–O bending vibrations.

**Figure 5.**
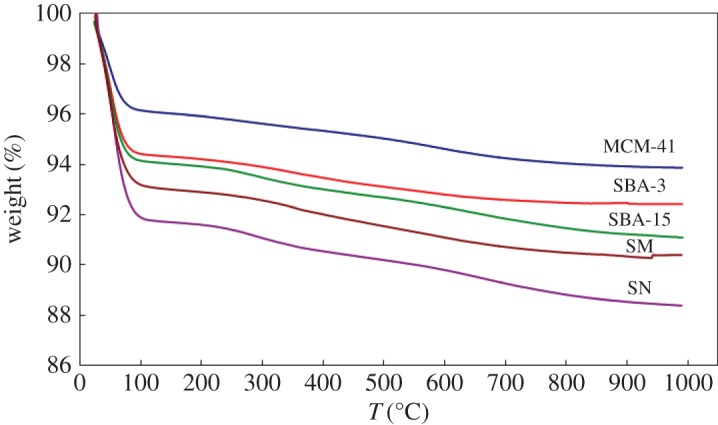
TGA of silica samples: MCM-41; SBA-3; SBA-15; SN and SM.

TGA of the prepared silica samples by different methods after calcination at 550°C for 6 h are shown in figure 5. According to the TG curves of silica samples prepared by different methods, they were found to exhibit similar weight loss behaviour. The weight loss of approximately 6–11% seems to occur below 200°C, most probably corresponding to the elimination of the absorbed water. There are no weight losses after that which confirm the complete removal of the surfactant template from the prepared silica materials during the calcination steps.

Table 2 shows that MCM-41, SBA-3 and SN were with smallest average aggregate particle sizes (56.0, 65.4 and 72.9 nm, respectively) among the prepared samples while the zeta potential values were the highest (−32.8, −46.1 and –31.4 mV, respectively). In comparison, SBA-15 and SM were with a wider distribution of particle size with an average of 220.9 and 260.7 nm and with the lowest zeta potential (−26.3 and −25.9 mV) among the prepared samples. Silica samples dispersed in water may be aggregated due to attractive van der Waals forces. By altering the dispersing conditions, repulsive forces can be introduced between the particles to eliminate these aggregates.

**Table 2.** Average aggregate size of SiO_2_ samples: MCM-41, SBA-3, SBA-15, SN and SM.

materials	MCM-41	SBA-3	SBA-15	SN	SM
Average aggregate size (nm)	55.97	65.36	220.9	72.92	260.7
Zeta potential (mV)	−32.8	−46.1	−26.3	−31.4	−25.9

**Figure 6.**
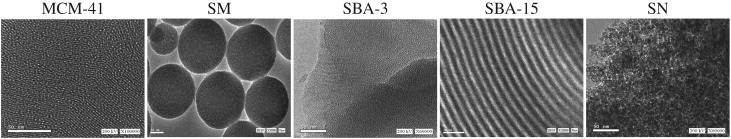
TEM images of silica samples: MCM-41; SM; SBA-3; SBA-15 and SN.

The structures of the silica samples can be directly observed by TEM. The TEM images in figure 6 (MCM-41, SBA-3 and SBA-15) illustrate the well-ordered hexagonal arrays of mesopores viewed along the channel direction, and the TEM images of SN show very fine, homogeneous and well-distributed nanoparticles. SM shows monodisperse spheres of 200 ± 10 nm.

### 3.2. Methylene blue uptake as an organic molecule model

#### 3.2.1. Effect of adsorbent dose

**Figure 7.**
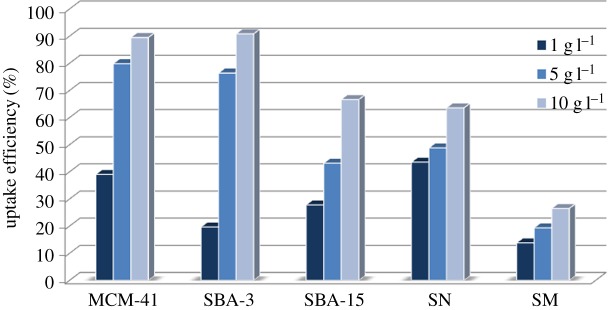
Effect of adsorbent doses of different silica materials on the MB uptake efficiency (keeping the concentration at 100 ppm and contact time of 60 min).

The optimum amount of the adsorbent required for quantitative removal of 100 ppm of MB was determined by investigating different adsorbent doses. Three different doses of each silica material such as 1, 5 and 10 g l^−1^ were compared, as shown in figure 7. Generally, the adsorption efficiency of MB is enhanced when the amount of materials used increased, which is attributed to the increased availability of adsorption sites. The sorption mechanism was mainly attributed to the huge silica surface area which results in absorption inside the mesopores besides enhanced interactions between the cationic dye molecules and negative charges on the silica surface as indicated from the zeta potential values.

Silica microparticles achieved the lowest adsorption efficiency for all tested doses due to their large particle size and the lowest surface area. SNs were the most efficient materials when using the smallest tested dose (1 g l^−1^), and this is due to the availability of all the adsorption sites and the easy accessibility of adsorption sites upon contact with MB dye molecules. By increasing the adsorbent dose to 5 and 10 g l^−1^, the uptake efficiency increased. MCM-41 and SBA-3 achieved the highest efficiency using doses of 5 and 10 g l^−1^.

**Figure 8.**
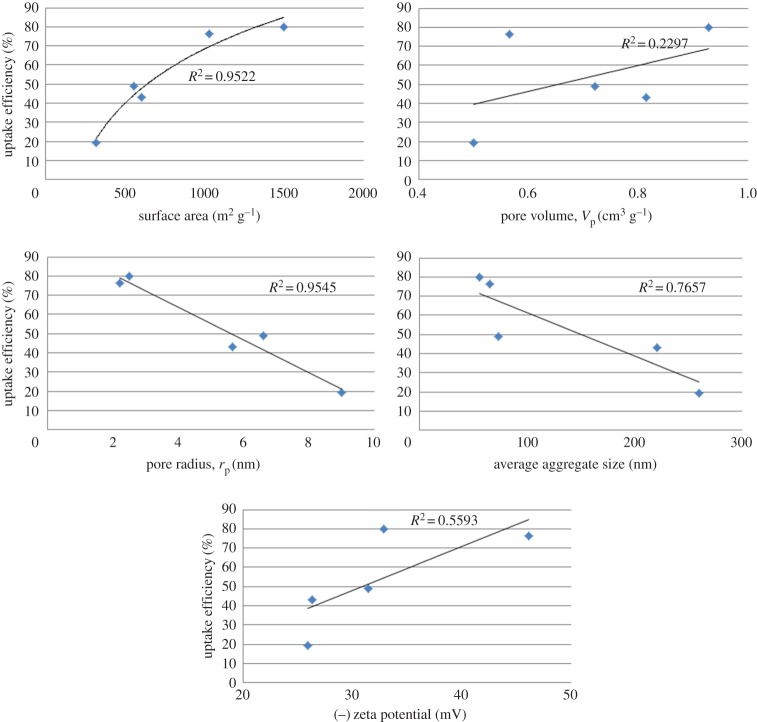
Correlation between the uptake capacity using 5 g l^−1^, 100 ppm for 60 min and the physical properties of the different prepared silica materials.

Correlations between the uptake capacities obtained using a dose of 5 g l^−1^ and different physical and surface properties of the different prepared silica types are represented in figure 8. Generally, there is no consensus between the researchers regarding the most effective factors controlling the adsorption process. The process is commonly related to the properties of the adsorbate species in their aqueous solution and the energy of surface binding and interactions in addition to the energy and availability of adsorbent sites. Another factor is the accessibility to adsorption centres, which also can be linked to the sizes of species to be adsorbed and their effective charge. Plotting the dependence of the maximum achieved uptake efficiency against the physical-surface properties of materials reveals linear trends as presented in figure 8. In our experiments, depending on the *R*^2^ values of relations in figure 8, it is interesting that the order of the amounts adsorbed at the same experimental conditions is directly agreed with the surface area and surface functional groups represented by the zeta potential values of the silica materials (which confirms the suggested sorption mechanism mainly attributed to both the huge surface area and the enhanced interactions between the cationic dye molecules and negative charges on the silica surface). The order is, on the other hand, inversely related to pore radius and average aggregate size. The pore volume was found to be the least dependent physical-surface property.

#### 3.2.2. Effect of contact time and the kinetic studies

**Figure 9.**
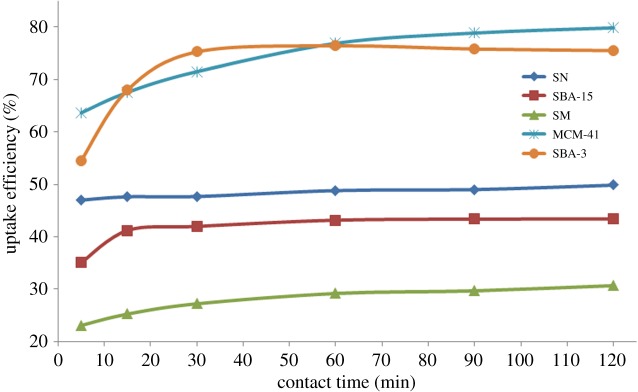
MB uptake efficiency of different silica materials over time (keeping the concentration at 100 ppm and adsorbent dose at 5 g l^−1^).

Different contact times were investigated from 5 up to 120 min in order to determine the equilibrium contact time, and the results are shown in figure 9. The tested materials were found to be differentiable regarding the contact time required to reach the equilibrium; SNs were the fastest to reach equilibrium within 5 min after which only a slight increase in the uptake efficiency can be observed. By contrast, SMs, with the highest average aggregate size, reached the equilibrium within 60 min. Regarding the ordered mesoporous silica types, SBA-15 reached the equilibrium within 15 min and SBA-3 within 30 min, while MCM-41 reached the equilibrium within 1 h with the maximum uptake efficiency among the tested silica materials. The difference in the equilibrium contact time confirms the suggested effect of different physical and surface properties on the accessibility and availability of adsorption sites.

For further analysis of the results, several kinetic models are used to describe the adsorption process. Four common equations were tested to find the best-fitted model for the experimental data [60,61].

The pseudo-first-order kinetic model for the adsorption of solid/liquid systems in its linear form can be formulated as

3.1 $$\text{ln}(q_{e}-q_{t})=\frac{\text{ln}(q_{e})-K_{1p}t}{2.303}.$$

The first-order kinetic model linear form can be formulated as

3.2 $$\text{log}\frac{q_{e}-q_{t}}{q_{e}}=\frac{K_{1}t}{2.303}.$$

Ho and McKay's pseudo-second-order kinetic model can be expressed as

3.3 $$\frac{t}{q_{t}}=\frac{1}{2k_{2p}q_{e}^{2}}+\frac{t}{q_{e}}.$$

The second-order kinetic model linear form can be formulated as

3.4 $$\frac{1}{q_{e-}q_{t}}=\frac{1}{q_{e}}+k_{2}t,$$

where *q*_e_ and *q*_t_ are the amount of dye adsorbed (mg g^−1^) at equilibrium and at time *t*, respectively. *k*_1p_ is the equilibrium rate constant of the pseudo-first-order adsorption (min^−1^). *k*_2p_ is the equilibrium rate constant of the pseudo-second-order adsorption (min^−1^). *k*_1_ is the equilibrium rate constant of the first-order adsorption (min^−1^). *k*_2_ is the equilibrium rate constant of the second-order adsorption (min^−1^).

**Figure 10.**
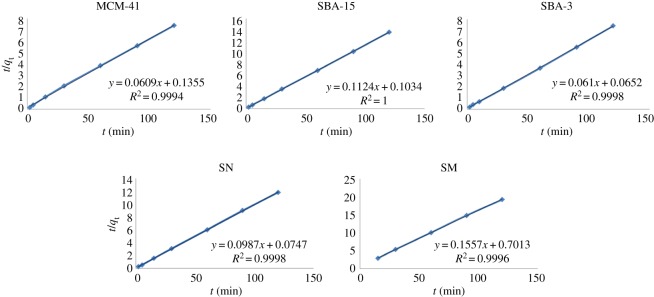
Adsorption kinetics pseudo-second-order model of MB dye molecules onto the prepared silica materials.

The experimental data have been fitted by the mentioned kinetics models. Based on the analysis of the *R*^2^ of the linear form for various kinetics models, the pseudo-second-order model was more appropriate to describe the adsorption kinetics behaviours for MB dye molecules onto all the prepared silica materials, indicating that the uptake process is controlled by chemical adsorption (chemisorption) in which it is assumed that the adsorption capacity is proportional to the number of active sites occupied on the adsorbent surface [62]. The fitting of the experimental data to the pseudo-second-order model is shown in figure 10.

#### 3.2.3. Effect of initial methylene blue concentration and the adsorption isotherms

**Figure 11.**
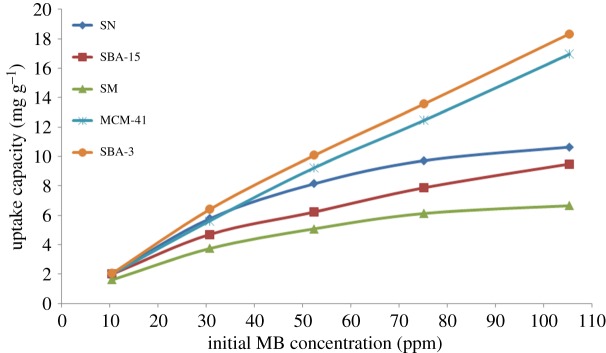
Effect of initial MB concentration on the uptake capacity of silica materials (keeping the contact time at 60 min and adsorbent dose at 5 g l^−1^).

The analysis of the adsorption process requires the relevant adsorption equilibrium, which is the most important piece of information in understanding an adsorption process. Adsorption equilibrium provides the fundamental physico-chemical data for evaluating the applicability of the adsorption process [63]. Figure 11 represents the relationship between the amount of MB adsorbed onto the adsorbent and the initial MB concentration in the aqueous phase. The adsorption capacity was found to increase with the initial MB concentration, progressively reaching a state of saturation of the adsorbent with barely increase in the uptake capacity in case of SN and SM, while the ordered mesoporous materials, MCM-41, SBA-3 and SBA-15, show a linear increase in uptake capacity with the increase in initial MB concentration.

For further analysis of the results, common isotherm equations were tested to find the best-fitted adsorption isotherm model for the obtained experimental data.

*Langmuir isotherm*: the experimental adsorption data were fitted according to the Langmuir isotherm model, with the following equation being preferentially used for studies on adsorption in solution [64].

3.5 $$q_{e}=\frac{K_{L}C_{e}q_{m}}{1+K_{L}C_{e}},$$

where *q*_e_ and *C*_e_ are the amount adsorbed (mg g^−1^) and the adsorbate concentration in solution (mg l^−1^), respectively, both at equilibrium. *K*_L_ is the Langmuir constant (l mg^−1^) and *q*_m_ is the maximum adsorption capacity of the monolayer formed on the adsorbent (mg g^−1^).

Langmuir model assumes that the adsorbent surface has sites of identical energy and that each adsorbate molecule is located at a single site; hence, it predicts the formation of a monolayer of the adsorbate on the adsorbent surface [65]. The linear form of the Langmuir isotherm is represented by the following equation:

3.6 $$\frac{C_{e}}{q_{e}}=\frac{1}{K_{L}q_{m}}+\frac{C_{e}}{q_{m}}.$$

Fitting of Langmuir isotherm suggests forming monolayer of adsorbed molecules on the material-binding sites.

Freundlich isotherm is an empirical equation employed to describe equilibrium on heterogeneous surfaces and hence does not assume monolayer capacity. Mathematically, it is expressed by

3.7 $$q_{e}=K_{f}C^{1/n}.$$

Equation (3.7) can also be expressed in the linearized logarithmic form as

3.8 $$\text{log}\;q_{e}=\text{log}\;K_{f}+\;\frac{1}{n}\log\;C_{e},$$

where *K*_f_ and *n* are the Freundlich isotherm constants indicating the adsorption capacity (mg g^−1^) and adsorption intensity (unitless), respectively [66,67]. Fitting of Freundlich isotherm suggests the presence of different binding sites in the investigated materials.

**Figure 12.**
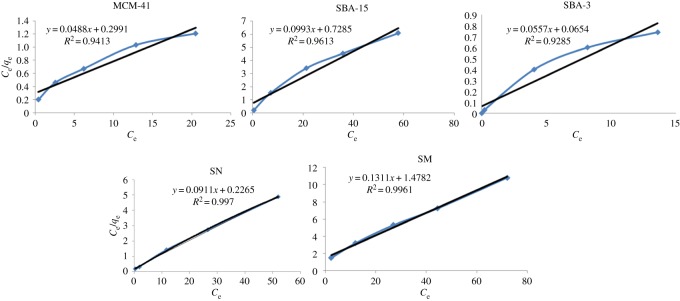
Langmuir adsorption isotherm fitting of silica materials.

**Figure 13.**
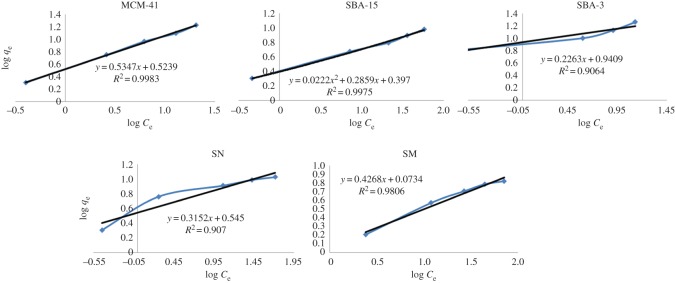
Freundlich adsorption isotherm fitting of silica materials.

On analysing the values of *R*^2^ obtained for the isotherm models (represented in figures 12 and 13), it can be observed that Langmuir equation provided the best fit for the experimental data in case of SN and SM, confirming thus uniform adsorption site, while Freundlich model provided the best fit for the experimental data in case of MCM-41, SBA-3 and SBA-15, confirming multi-positions of adsorption; the outer functional surface of silica particles and the ordered inner mesopores.

#### 3.2.4. Flow system treatment

**Figure 14.**
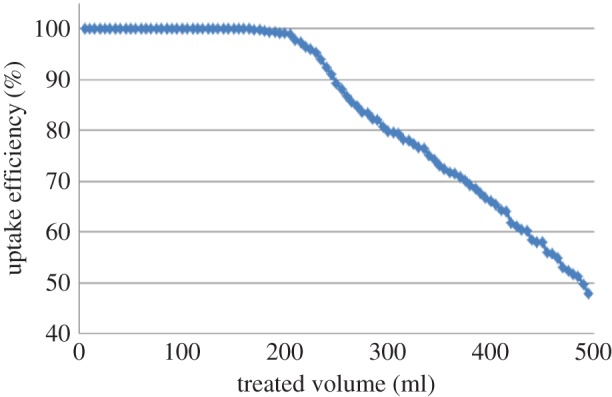
Flow system uptake using MCM-41.

MB loading was carried out also using a column containing 1 g of MCM-41. Up to 200 ml of the treated solution gives pure water with complete adsorption of MB after that, the treated solution gives slightly coloured water with low concentration of MB which increases with the increase of the treated solution volume as shown in figure 14.

Future work involving further studies of these materials as molecular species carrier, and loading and release behaviours will be done to investigate the applicability of these materials as drug-controlled release and smart container materials.

## 4. Conclusion

A simple eco-friendly preparation of silica materials such as MCM-41, SBA-3, SBA-15, SN and SM by sol–gel method gives rise to unique material series of huge surface area with mesoporous characteristics. It can be concluded that upon using the same starting material, a variety of materials can be prepared using different preparation conditions such as media pH, a surfactant template and the type of this surfactant. The results confirm the applicability of the silica nanomaterials in effective filtration and purification systems.

## Supplementary Material

Attached to the submitted files in the name "Data"

## Data accessibility

The data of this work are available in the electronic supplementary material.
